# Playing basketball and volleyball during adolescence is associated with higher bone mineral density in old age: the Bunkyo Health Study

**DOI:** 10.3389/fphys.2023.1227639

**Published:** 2023-10-12

**Authors:** Hikaru Otsuka, Hiroki Tabata, Huicong Shi, Mari Sugimoto, Hideyoshi Kaga, Yuki Someya, Hitoshi Naito, Naoaki Ito, Abulaiti Abudurezake, Futaba Umemura, Tsubasa Tajima, Saori Kakehi, Yasuyo Yoshizawa, Muneaki Ishijima, Ryuzo Kawamori, Hirotaka Watada, Yoshifumi Tamura

**Affiliations:** ^1^ Sportology Center, Graduate School of Medicine, Juntendo University, Bunkyo-ku, Tokyo, Japan; ^2^ Department of Sports Medicine and Sportology, Graduate School of Medicine, Juntendo University, Bunkyo-ku, Tokyo, Japan; ^3^ Department of Metabolism and Endocrinology, Graduate School of Medicine, Juntendo University, Bunkyo-ku, Tokyo, Japan; ^4^ Graduate School of Health and Sports Science, Juntendo University, Inzai-shi, Chiba, Japan; ^5^ Department of Healthy Life Expectancy, Graduate School of Medicine, Juntendo University, Bunkyo-ku, Tokyo, Japan; ^6^ Department of Medicine for Orthopaedics and Motor Organ, Graduate School of Medicine, Juntendo University, Bunkyo-ku, Tokyo, Japan; ^7^ Faculty of International Liberal Arts, Juntendo University, Bunkyo-ku, Tokyo, Japan

**Keywords:** bone mass, sports type, cross-sectional study, femoral neck, lumbar spine, exercise history

## Abstract

**Introduction:** Exercise is beneficial for increasing areal bone mineral density (aBMD) in adolescence and maintaining it in old age. Moreover, high-impact sports are more effective than low-impact sports in increasing aBMD. This study aimed to determine the types of adolescent sports played in school-based sports clubs associated with aBMD in old age.

**Methods:** In total, 1,596 older adults (681 men and 915 women, age: 65–84 years) living in an urban area of Japan were evaluated for the femoral neck and lumbar spine aBMD using dual-energy X-ray absorptiometry. The association between adolescent sports played in sports clubs and aBMD in old age was analyzed using multiple regression analysis, with femoral neck and lumbar spine aBMD as dependent variables, and sports type and participant characteristics such as age, body weight, and serum 25-hydroxyvitamin D [25(OH)D] level, as independent variables.

**Results:** For the femoral neck, basketball was associated with aBMD in older men (*β* = 0.079, *p* < 0.05) and women (*β* = 0.08, *p* < 0.01), whereas current body weight and 25(OH)D level were associated with aBMD in both sexes. For the lumbar spine, volleyball (*β* = 0.08, *p* < 0.01) and swimming (*β* = 0.06, *p* < 0.05) was significantly associated with lumbar spine aBMD, whereas current body weight, 25(OH)D, and diabetes mellitus were associated with aBMD in older women.

**Conclusion:** Both men and women who played basketball in adolescence had higher femoral neck aBMD in old age. Moreover, women who played volleyball in adolescence had higher lumbar spine aBMD in old age.

## Introduction

Osteoporosis is a systemic skeletal disease characterized by low bone mass (BM) and microarchitectural deterioration of bone tissue, with a consequent increase in bone fragility and susceptibility to fracture (Peck et al., 1993; Johnell and Kanis, 2005). Since osteoporosis increases the risk of long-term care and healthcare costs, prevention is crucial (Mori et al., 2022). BM significantly increases during adolescence, reaching peak bone mass (PBM) in the 20’s that is maintained until around 50 years, after which it decreases with age (Hendrickx et al., 2015). According to a study, a 10% increase in PBM delays the onset of osteoporosis by 13 years (Rizzoli et al., 2010). Accordingly, it is important to increase the PBM during adolescence to prevent osteoporosis.

Adolescence is a key period for bone accrual. Depending on the skeletal site, about 40% adult bone mass is determined during adolescence (Baxter-Jones et al., 2011). Exercise during adolescence is effective in increasing PBM (Karlsson and Rosengren, 2020; Zhu and Zheng, 2021) because it generates the biomechanical stimulus required to increase BM (Singh, 2004; Kalabiska et al., 2020; Maillane-Vanegas et al., 2020). In general, when muscles contract during exercise, they generate mechanical forces that are transmitted to the bones, which stimulates bone growth and remodeling (Burr, 1997). Therefore, the effect of exercise on BM depends on the biomechanical stimulus of each exercise (Parfitt, 1994; Turner and Robling, 2003; Tenforde and Fredericson, 2011). For example, in comparison to non-impact sports such as swimming and cycling, high-impact (e.g., basketball, gymnastics, hurdling, judo, karate, volleyball, and other jumping sports) and odd-impact sports (e.g., soccer, racquet games, step aerobics, and speed skating) are highly associated with high bone mineral composition, areal bone mineral density (aBMD), and bone geometry in adolescence (Agostinete et al., 2020; Maillane-Vanegas et al., 2020).

Since PBM gained at a young age predicts approximately 50% of the bone mass variation in old age (Hui et al., 1990), those who engage in exercise have more likelihood of higher aBMD in the later years. For example, former male athletes who played soccer in their youth maintained higher aBMD and had a reduced risk of fragility fractures after 30 years of age (Tveit et al., 2015). In addition, women athletes who performed gymnastics before menarche maintained a high bone mass 10 years after they stopped training (Erlandson et al., 2012). It remains unclear whether similar long-term effects can be gained from club sports activities among non-athletes. Furthermore, although high-impact sports are more effective in increasing aBMD than low-impact sports, it remains unknown which types of adolescent sports are associated with a high aBMD in old age. The purpose of the present study was to examine the association between the type of sports played in adolescence and aBMD in old age. We hypothesized that participation in relatively high-impact adolescent exercises in a sports club would be associated with higher aBMD in old age, even in non-athletes.

## Materials and methods

### Study design and participants

This cross-sectional study used the baseline data from the Bunkyo Health Study (Someya et al., 2019; Otsuka et al., 2021). The Bunkyo Health Study recruited adults aged 65–84 years living in Bunkyo-Ku, an urban area in Tokyo, Japan. Bunkyo-ku was chosen because the age distribution is similar to that of Tokyo as a whole. All participants completed the 2-day examinations at the Sportology Center from 15 October 2015 to 1 October 2018. Briefly, we evaluated physical fitness using dynamometry and physical performance tests, body composition, blood biochemical markers, and aBMD using dual-energy X-ray absorptiometry (DXA). The study protocol was approved by the ethics committee of Juntendo University in November 2015 (approval number: 2015078; latest revised version number: 2021095) and was conducted in accordance with the principles of the Declaration of Helsinki. All the participants provided written informed consent and were notified that they had the right to withdraw from the trial at any time.

**FIGURE 1:**
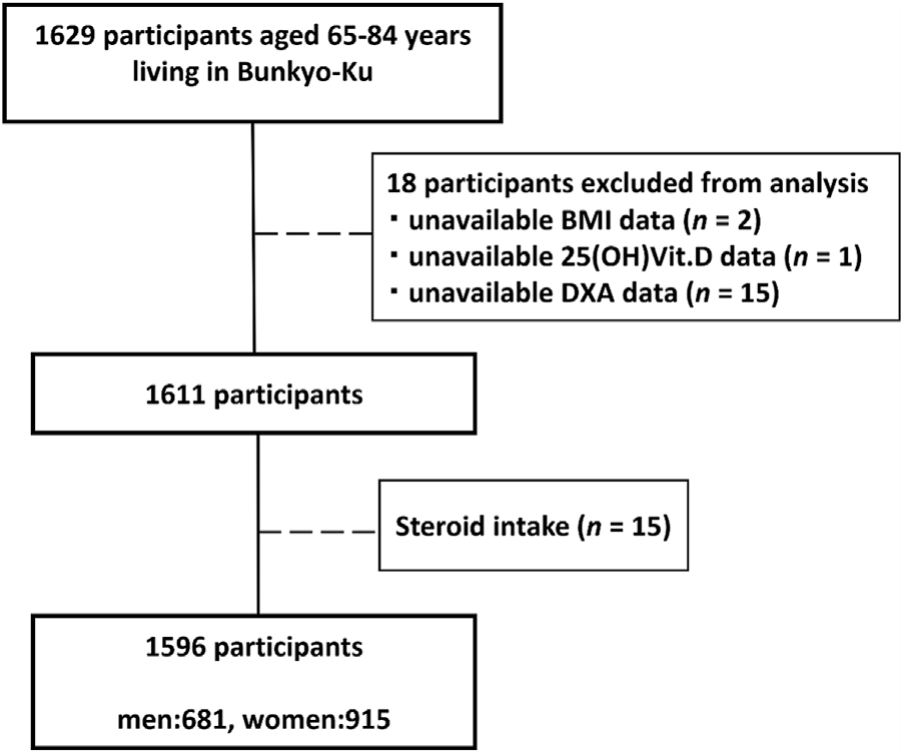
Flowchart of the participants. Among 1,629 participants enrolled in the Bunkyo Health Study, we excluded 18 with unavailable data [BMI (*n* = 2), 25-hydroxyvitamin D [25(OH)D] (*n* = 1), and DXA (*n* = 15)]. Furthermore, of the remaining 1,611 participants, 15 who received corticosteroids for the treatment of each disease were also excluded. Finally, 1,596 participants (men: 681 and women: 915) were included in this analysis.

Among 1,629 participants enrolled in the Bunkyo Health Study, we excluded 18 with unavailable data [BMI (*n* = 2), 25-hydroxyvitamin D [25(OH)D] (*n* = 1), and DXA (*n* = 15)]. Furthermore, of the remaining 1,611 participants, 15 who received corticosteroids for the treatment of each disease were also excluded. Finally, 1,596 participants (men: 681 and women: 915) were included in this analysis (Figure 1).

### Bone mineral density measurements

The aBMD of the femoral neck and lumbar spine (L2-L4) was measured using the Discovery DXA System (Hologic Inc., Massachusetts, United States). Positioning and image acquisition were performed according to the International Society for Clinical Densitometry (ISCD) protocol, measuring L2-L4 for lumbar spine ROI and femoral neck for hip ROI (Lewiecki et al., 2004). Quality of the longitudinal evaluation was assured by calibrating the machine with standardized phantoms. aBMD is expressed as standard deviation (SD) units relative to the aBMD of young persons (T-score). For quality control, daily scans were performed before and after study measurements using a site-specific lumbar phantom (Bazzocchi et al., 2016). The coefficient of variation (CV) was determined to be 0.4% using the measurement values from the lumbar phantom.

### Sport types

An interview was conducted to investigate the status of exercise implementation among adolescents. We defined those who answered “yes” to the question “Did you participate in “Bukatsudo” when you were in junior high school or high school” as having exercise habits during adolescence, and we also asked them what types of sports they were involved in. In Japan, school-based sports club activities are referred to as “Bukatsudo.” While participation in Bukatsudo is not mandatory, they are integrated into the educational framework. Given that these activities receive financial support from schools, the economic burden on participants is diminished (Cave, 2004; Japan Sports Agency, 2018). As a result, approximately 90% of Japanese adolescents in junior high schools (12–15 years old) and high schools (15–18 years old) who engage in physical exercise do so in Bukatsudo (Sasagawa sports foundation, 2019). In general, the students enrolled in a Bukatsudo practice three to three and a half hours daily, 6 days a week, regardless of school vacations; thus, sports clubs are a major part of their school life (Cave, 2004). For this reason, it is assumed that there is limited recall bias. In the present study, participants engaged in 31 types of sports (e.g., soccer, swimming, tennis, basketball, volleyball, baseball, and track and field) in their junior high and high school. Therefore, we categorized sports types into five groups based on level of bone loading in previous studies: Non-sports: those who were not involved in Bukatsudo in junior high or high school; Repetitive non-impact: muscle forces during long-lasting performances without ground impact, such as swimming, kyudo, rowing, golf, equestrian, aviation, and sailing; Repetitive low-impact: ground impacts during long-lasting performances at a relatively constant speed such as table tennis, mountaineering, ski and skating, and dance; Odd-impact: involves rapid turns and stops with ground impacts such as baseball and softball, judo, tennis, track and field, soccer, kendo, rugby, boxing, karate, handball, wrestling, ice hockey, badminton, weightlifting, and American football; High-impact: includes maximal vertical jumps and ground impacts such as basketball, volleyball, gymnastics. Some participants performed multiple sports, in which case we selected the higher-impact sports (Nikander et al., 2010; Hervás et al., 2019). Among the 31 sports, “skiing and skating” and “baseball and softball” were combined into one sport as similar types. Consequently, 29 types of sports were included in this analysis.

### Other measurements

Physicians interviewed participants using a structured questionnaire to assess their medical history and current medication status. The questionnaire included questions regarding the presence of age-related diseases, such as type 2 diabetes and osteoporosis, as well as current medications. Self-administered questionnaires were used to determine the following: sex (men or women), age (in years), and smoking status (current and former smoking history). Current physical activity level was evaluated using the International Physical Activity Questionnaire (IPAQ) (Bassett, 2003; Murase, 2003). Also, we asked, “Did you have exercise habits at each age from your 20’s to 50’s?”, those who responded “yes” were defined as having exercise habits at each age from their 20’s to 50’s. Dietary intake was assessed using a brief self-administered diet history questionnaire (BDHQ) to measure alcohol intake. The BDHQ had been validated in previous studies (Kobayashi et al., 2011; Kobayashi et al., 2012). Blood samples were collected in the morning to measure biochemical markers after an overnight fast. Serum 25(OH)D concentrations were analyzed using a chemiluminescent enzyme immunoassay. Diabetes mellitus was defined as fasting blood glucose ≥126 mg/dL, 2-h blood glucose level ≥200 mg/dL after a 75-g oral glucose tolerance test, hemoglobin A1c ≥ 6.5%, or current use of diabetes medication. All blood samples were tested at the commissioned clinical laboratory center (SRL Inc., Tokyo, Japan).

### Equity, diversity, and inclusion statement

The target population consisted of only older Japanese adults living in Bunkyo-Ku, Tokyo. The sample population was selected from among 68 communities in Bunkyo-Ku; we selected 13 communities based on probability proportional to size sampling. We obtained the names and addresses of all residents aged 65–84 years in the selected communities from residential registries and sent invitation letters requesting participation in this study.

### Statistical analysis

The characteristics of sex differences were compared using Mann-Whitney U tests and chi-square tests for continuous and categorical variables, respectively. The continuous variables are presented as the median with their interquartile range, whereas the categorical variables are shown as frequencies and percentages. We conducted an analysis of covariance (ANCOVA) to investigate the relationships between the five groups and BMD (femoral neck and lumbar spine). The participants were categorized into the following five groups based on the level of bone loading: Non-sports, Repetitive non-impact, Repetitive low-impact, Odd-impact, High-impact. The potential confounders were age (continuous variable), body weight (continuous variable), calcium intake (continuous variable), alcohol intake (continuous variable), 25(OH)D level (continuous variable), years of education (continuous variable), current physical activity level (continuous variable), current and past smoking status (yes or no), diabetes (yes or no), taking osteoporosis drugs or estrogens (yes or no). These were reported as the mean and standard errors. Those who had engaged in multiple sports were adopted for the higher impact sport. When significant differences were found, the Bonferroni test was used for multiple comparisons between groups. Subsequently, the association between adolescent sports type and aBMD in old age was analyzed using multiple regression analysis. In this analysis, femoral neck and lumbar spine aBMD were used as dependent variables, and sports type, physical characteristics, and biochemical parameters were used as independent variables. In the regression analysis, we adjusted for potential confounders associated with aBMD; age (continuous variable), body weight (continuous variable), alcohol intake (continuous variable), 25(OH)D (continuous variable), current physical activity level (continuous variable), calcium intake (continuous variable), years of education (continuous variable), diabetes mellitus (yes or no), taking osteoporosis drugs or estrogens (yes or no) and current and past smoking history (yes or no). If the participants had engaged in multiple sports during junior or high school, for the covariance analysis where we divided sports into five groups, we classified them based on the sport with the highest level of bone loading. However, in the multiple regression analysis, participants who engaged in multiple sports were accounted for as participating in each respective sport. Additionally, for statistical power considerations, we excluded sports with fewer than 10 participants. Significantly between men and women, we analyzed the data separately. We did not center the continuous variables prior to their inclusion in the regression models. Consequently, the reported beta values directly represent the raw score associations. Statistical Package for the Social Sciences v. 27.0 for Windows (SPSS Inc. Released 2008. SPSS Statistics for Windows, Version 27.0. Chicago: SPSS Inc.) was used to analyze the data. All statistical tests were two-sided with a significance level of 5%.

## Results

### Characteristics of the men and women participants

The characteristics of the men and women participants are shown in Table 1. In total, 428 (62.8%) men and 429 (46.9%) women participated in junior or high school club activities. Both femoral neck aBMD and lumbar spine aBMD were within normal ranges for the men, whereas the women showed low values at both sites. The number of women on osteoporosis medication was significantly higher than that of men (14.3% vs. 0.7%). In contrast, diabetes mellitus, physical activity, and current smoking were more prevalent among men and alcohol intake was approximately six times higher than among women.

**TABLE 1:** Characteristics of the participants in old age.

	Men	Women	*p*-value
Number (n/%)	681 (42.7)	915 (57.3)	
Sports club activities in adolescent (n/%)	428 (62.8)	429 (46.9)	*p* < 0.001
Age (years)	73 (68–77)	73 (68–77)	*p* = 0.698
Height (cm)	165.8 (161.5–170.0)	152.5 (149.0–156.0)	*p* < 0.001
Bodyweight (kg)	65.4 (59.6–71.1)	52.2 (47.3–57.2)	*p* < 0.001
BMI (kg/m^2^)	23.7 (22.1–25.6)	22.5 (20.5–24.6)	*p* < 0.001
Body fat (%)	17.9 (15.2–20.8)	26.2 (22.5–29.6)	*p* < 0.001
Calcium intake (mg/day)	635.6 (488.3–844.1)	680.1 (508.5–876.2)	*p* = 0.008
Alcohol intake (g/day)	13.0 (0.3–36.9)	0.2 (0–3.5)	*p* < 0.001
25(OH)D (nmol/L)	19.5 (16–23)	18.0 (15–21)	*p* < 0.001
Current physical activity level (Mets·hour/week)	32.5 (18.6–57.3)	29.2 (16.5–51.6)	*p* = 0.026
Current smoking (n/%)	90 (13.2)	30 (3.3)	*p* < 0.001
Smoking history (n/%)	497 (73.0)	160 (17.5)	*p* < 0.001
Diabetes mellitus (n/%)	123 (18.1)	81 (8.9)	*p* < 0.001
Years of education (n/%)	16 (12–16)	12 (12–14)	*p* < 0.001
Femoral neck (g/cm^2^)	0.728 (0.654–0.808)	0.576 (0.52–0.642)	*p* < 0.001
Lumbar spine (g/cm^2^)	1.048 (0.923–1.196)	0.811 (0.715–0.932)	*p* < 0.001
Taking osteoporosis medication (n/%)	5 (0.7)	131 (14.3)	*p* < 0.001

### Association between adolescent sports and aBMD in old age

The types of adolescent sports in this cohort are presented in Supplementary Table S1. The major sports among men were baseball/softball (*n* = 99), basketball (*n* = 65), judo (*n* = 49), table tennis (*n* = 46), and tennis (*n* = 45). In contrast, the major sports among women were volleyball (*n* = 148), tennis (*n* = 87), table tennis (*n* = 72), basketball (*n* = 53), and baseball/softball (*n* = 38). A comparison of femoral neck and lumbar spine aBMD between the five groups divided by level of bone loading is shown in Table 2. ANCOVA results showed that there was no significantly different aBMD at the femoral neck (men: *p* = 0.853, women: *p* = 0.687) and lumbar spine (men: *p* = 0.097, women: *p* = 0.245) for both men and women. The associations between adolescent sport types and aBMD in older age groups are shown in Tables 3–6. Basketball was significantly associated with a high femoral neck aBMD in both older men (Table 3; *β* = 0.079, *p* = 0.033) and women (Table 4; *β* = 0.080, *p* = 0.008). Additionally, body weight (*β* = 0.301, *p* = 0.000), alcohol intake (*β* = 0.123, *p* = 0.001), and 25(OH)D (*β* = 0.097, *p* = 0.009) level in older men and body weight (*β* = 0.380, *p* = 0.000) and 25(OH)D (*β* = 0.121, *p* = 0.000) in older women were significantly associated with a high femoral neck aBMD. In older men (Table 5), there was no association between adolescent sports type and lumbar spine aBMD, whereas body weight (*β* = 0.291, *p* = 0.000), age (*β* = 0.109, *p* = 0.006), and alcohol intake (*β* = 0.097, *p* = 0.011) were associated with lumbar spine aBMD. In contrast, in older women (Table 6), volleyball (*β* = 0.080, *p* = 0.009) and swimming (*β* = 0.060, *p* = 0.050) was significantly associated with lumbar spine aBMD, whereas body weight (*β* = 0.365, *p* = 0.000), 25(OH)D (*β* = 0.143, *p* = 0.000) level, and diabetes mellitus (*β* = 0.084, *p* = 0.006) were significantly associated with lumbar spine aBMD.

**TABLE 2:** Comparison of aBMD across different levels of bone loading exercise groups.

Men	Non-sports	Repetitive non-impact	Repetitive low-impact	Odd-impact	High-impact	*p*-value
	*n* = 308	*n* = 28	*n* = 32	*n* = 265	*n* = 48	
Femoral neck aBMD	0.732 ± 0.006	0.726 ± 0.02	0.741 ± 0.018	0.734 ± 0.006	0.718 ± 0.015	*p* = 0.853
Lumber spine aBMD	1.047 ± 0.011	1.102 ± 0.036	1.065 ± 0.033	1.089 ± 0.012	1.059 ± 0.027	*p* = 0.097
Women	Non-sports	Repetitive non-impact	Repetitive low-impact	Odd-impact	High-impact	*p*-value
	*n* = 537	*n* = 13	*n* = 54	*n* = 133	*n* = 177	
Femoral neck aBMD	0.582 ± 0.004	0.587 ± 0.024	0.598 ± 0.012	0.587 ± 0.007	0.588 ± 0.006	*p* = 0.687
Lumber spine aBMD	0.823 ± 0.006	0.833 ± 0.042	0.838 ± 0.02	0.823 ± 0.013	0.852 ± 0.011	*p* = 0.245

Values are the means ± SE. Adjusted variables: Age, Body weight, smoking history (current and past), alcohol intake, calcium intake, 25(OH)D, presence of diabetes, Current physical activity level, years of education and taking osteoporosis drugs or estrogens.

**TABLE 3:** Associations between sports engaged in adolescence and femoral neck aBMD in older men.

Variables	Unadjusted *β*	Adjusted *β*	*p*-value	Adjusted *β* 95%CI	
				Lower	Upper
Basketball	0.030	0.079	0.033	0.002	0.057
Tennis	0.030	0.067	0.072	−0.003	0.062
Rugby	0.049	0.059	0.109	−0.011	0.110
Soccer	0.009	0.014	0.700	−0.035	0.052
Table tennis	0.004	0.009	0.816	−0.028	0.035
Baseball and softball	0.002	0.006	0.880	−0.021	0.025
Track and field	−0.003	−0.006	0.875	−0.036	0.031
Judo	−0.003	−0.006	0.865	−0.034	0.028
Swimming	−0.003	−0.006	0.861	−0.043	0.036
Mountaineering	−0.004	−0.007	0.853	−0.051	0.043
Volleyball	−0.008	−0.016	0.659	−0.043	0.027
Kendo	−0.023	−0.030	0.412	−0.077	0.031
Gymnastics	−0.036	−0.041	0.263	−0.099	0.027
Age (in years)	−0.001	−0.049	0.204	−0.003	0.001
Bodyweight (kg)	0.004	0.301	<0.001	0.003	0.005
Current smoking (n/%)	−0.016	−0.048	0.202	−0.040	0.008
Past Smoking (n/%)	0.001	0.006	0.880	−0.017	0.020
Years of education (n/%)	0.001	0.021	0.582	−0.002	0.004
Diabetes mellitus (n/%)	0.008	0.027	0.468	−0.013	0.028
Calcium intake (mg/day)	−9.72 × 10^−6^	−0.025	0.509	−3.86 × 10^−5^	1.91 × 10^−5^
Alcohol intake (g/day)	4.66 × 10^−4^	0.123	0.001	1.88 × 10^−4^	0.001
25(OH)D (nmol/L)	0.002	0.097	0.009	0.001	0.004
Taking osteoporosis medication (n/%)	−0.070	−0.054	0.134	−0.162	0.022
Current physical activity level (Mets·hour/week)	−1.03 × 10^−5^	−0.005	0.888	−1.54 × 10^−4^	1.34 × 10^−4^
					Adjusted *R* ^2^ value 0.124

**TABLE 4:** Associations between sports engaged in adolescence and femoral neck aBMD in older women.

Variables	Unadjusted *β*	Adjusted *β*	*p*-value	Adjusted *β* 95%CI	
				Lower	Upper
Basketball	0.033	0.080	0.008	0.008	0.057
Table tennis	0.010	0.028	0.350	−0.011	0.030
Track and field	0.013	0.025	0.399	−0.018	0.045
Baseball and softball	0.012	0.025	0.403	−0.016	0.040
Volleyball	0.006	0.023	0.442	−0.009	0.021
Tennis	0.006	0.018	0.555	−0.013	0.025
Dance	0.011	0.014	0.650	−0.036	0.058
Swimming	0.002	0.003	0.919	−0.041	0.045
Gymnastics	1.20 × 10^−4^	2.31 × 10^−4^	0.994	−0.030	0.031
Mountaineering	−3.33 × 10^−4^	−4.58 × 10^−4^	0.988	−0.043	0.042
Age (in years)	−0.003	−0.199	<0.001	−0.005	−0.002
Bodyweight (kg)	0.005	0.380	<0.001	0.004	0.005
Current smoking (n/%)	0.014	0.027	0.413	−0.020	0.049
Past Smoking (n/%)	−0.014	−0.055	0.103	−0.030	0.003
Years of education (n/%)	1.97 × 10^−4^	0.005	0.885	−0.002	0.003
Diabetes mellitus (n/%)	0.005	0.014	0.642	−0.015	0.025
Calcium intake (mg/day)	−1.24 × 10^−6^	−0.004	0.897	−2.01 × 10^−5^	1.76 × 10^−5^
Alcohol intake (g/day)	1.09 × 10^−4^	0.015	0.622	−3.25 × 10^−4^	0.001
25(OH)D (nmol/L)	0.002	0.121	<0.001	0.001	0.003
Taking osteoporosis medication (n/%)	−0.001	−0.004	0.898	−0.017	0.015
Current physical activity level (Mets·hour/week)	2.80 × 10^−6^	0.001	0.967	−1.31 × 10^−4^	1.36 × 10^−4^
					Adjusted *R* ^2^ value 0.206

**TABLE 5:** Associations between sports engaged in adolescence and lumbar spine aBMD in older men.

Variables	Unadjusted *β*	Adjusted *β*	*p*-value	Adjusted *β* 95%CI	
				Lower	Upper
Tennis	0.051	0.063	0.091	−0.008	0.109
Rugby	0.088	0.058	0.118	−0.022	0.197
Baseball and softball	0.028	0.050	0.190	−0.014	0.070
Judo	0.034	0.044	0.240	−0.023	0.090
Basketball	0.028	0.041	0.275	−0.022	0.077
Swimming	0.037	0.037	0.313	−0.035	0.108
Gymnastics	0.039	0.025	0.505	−0.076	0.153
Kendo	0.018	0.013	0.725	−0.081	0.116
Soccer	0.013	0.012	0.752	−0.067	0.093
Mountaineering	0.011	0.009	0.803	−0.075	0.097
Track and field	−0.002	−0.002	0.952	−0.062	0.059
Table tennis	−0.013	−0.016	0.662	−0.070	0.045
Volleyball	−0.014	−0.016	0.659	−0.078	0.049
Age (in years)	0.004	0.109	0.006	0.001	0.007
Bodyweight (kg)	0.007	0.291	<0.001	0.005	0.008
Current smoking (n/%)	−0.038	−0.065	0.092	−0.082	0.006
Past Smoking (n/%)	0.018	0.039	0.307	−0.016	0.052
Years of education (n/%)	−0.003	−0.034	0.369	−0.009	0.003
Diabetes mellitus (n/%)	0.032	0.062	0.099	−0.006	0.070
Calcium intake (mg/day)	−8.66 × 10^−6^	−0.012	0.747	−6.13 × 10^−5^	4.40 × 10^−5^
Alcohol intake (g/day)	0.001	0.097	0.011	1.53 × 10^−4^	0.001
25(OH)D (nmol/L)	0.001	0.025	0.511	−0.002	0.004
Taking osteoporosis medication (n/%)	−0.102	−0.044	0.231	−0.270	0.065
Current physical activity level (Mets·hour/week)	−5.33 × 10^−5^	−0.015	0.691	−3.16 × 10^−4^	2.09 × 10^−4^
					Adjusted *R* ^2^ value 0.103

**TABLE 6:** Associations between sports engaged in adolescence and lumbar spine aBMD in older women.

Variables	Unadjusted *β*	Adjusted *β*	*p*-value	Adjusted *β* 95%CI	
				Lower	Upper
Volleyball	0.035	0.080	0.009	0.009	0.062
Swimming	0.075	0.060	0.050	0.000	0.149
Basketball	0.025	0.036	0.244	−0.017	0.067
Table tennis	0.012	0.019	0.525	−0.024	0.047
Dance	0.018	0.013	0.666	−0.064	0.100
Tennis	0.007	0.013	0.670	−0.026	0.040
Baseball and softball	0.007	0.008	0.784	−0.042	0.055
Gymnastics	−0.001	−0.001	0.963	−0.054	0.052
Track and field	−0.014	−0.015	0.616	−0.068	0.041
Mountaineering	−0.048	−0.039	0.206	−0.122	0.026
Age (in years)	0.001	0.036	0.275	−0.001	0.003
Bodyweight (kg)	0.008	0.365	<0.001	0.006	0.009
Current smoking (n/%)	−0.027	−0.030	0.372	−0.087	0.033
Past Smoking (n/%)	−0.011	−0.027	0.437	−0.040	0.017
Years of education (n/%)	0.003	0.041	0.206	−0.002	0.008
Diabetes mellitus (n/%)	0.048	0.084	0.006	0.013	0.082
Calcium intake (mg/day)	−1.34 × 10^−5^	−0.025	0.421	−4.62 × 10^−5^	−1.93 × 10^−5^
Alcohol intake (g/day)	−4.01 × 10^−5^	−0.003	0.917	−0.001	0.001
25(OH)D (nmol/L)	0.005	0.143	<0.001	0.003	0.007
Taking osteoporosis medication (n/%)	−0.012	−0.026	0.413	−0.040	0.017
Current physical activity level (Mets·hour/week)	−1.24 × 10^−4^	−0.032	0.294	−3.57 × 10^−4^	1.08 × 10^−4^
					Adjusted *R* ^2^ value 0.168

Furthermore, we conducted a preliminary analysis adjusting for the young adult to middle-aged exercise habits score (Supplementary Tables S2–S6). The young adult to middle-aged exercise habits score was calculated according to having exercise habits in the 20’s to 50’s: score plus 1 for having an exercise habit and 0 for not having an exercise habit of each age period. ANCOVA results for a comparison of femoral neck and lumbar spine aBMD between the five groups divided by level of bone loading showed that there was no significantly different aBMD at the femoral neck (men: *p* = 0.897, women: *p* = 0.817) and lumbar spine (men: *p* = 0.172, women: *p* = 0.286) for both men and women (Supplementary Table S2). In a multiple regression analysis, basketball was significantly associated with a high femoral neck aBMD in both older men (Supplementary Table S3; *β* = 0.077, *p* = 0.037) and women (Supplementary Table S4; *β* = 0.075, *p* = 0.012). In older men (Supplementary Table S5), there was no association between adolescent sports type and lumbar spine aBMD. On the other hand, in older women (Supplementary Table S6), volleyball (*β* = 0.080, *p* = 0.010) was significantly associated with lumbar spine aBMD.

## Discussion

It remains unclear whether various adolescent sports activities are associated with higher aBMD later in life. In this study, we investigated the association between the type of sports played in school-based sports clubs (during junior high and high school) and aBMD in old age. As a result, there were no significant associations when categorizing the five groups by impact level. However, when focusing on a single sport, both men and women who played adolescent basketball in clubs had higher femoral neck aBMD in old age. In addition, women who played adolescent volleyball clubs had higher lumbar spine aBMD in old age. The innovation of this study is that sports in adolescence have a long-term effect on bone mineral density in old age, even in the general population, not just in athletes.

Adolescent participation in high-impact sports, such as basketball and volleyball, may enhance aBMD in later life. The novel aspect of this study suggests that exercise undertaken during the growth phase 50/60 years ago may have implications for bone mineral density in old age. High-impact sports involving jumping, such as basketball and volleyball, are known to increase PBM in adolescents (Carbuhn et al., 2010). Additionally, in recent meta-analysis especially basketball is effective in bone growth during adolescence (Stojanović et al., 2020). A modest cohort study involving 46 postmenopausal middle-aged women demonstrated that high-impact sports like basketball and volleyball in adolescence effectively preserved bone mineral content in the hip and lumbar spine in middle-aged (Kato et al., 2009). Furthermore, former male soccer players who were active in their youth and early adulthood exhibited a higher femoral neck aBMD in later years compared to controls who did not engage in exercise during the same timeframe (Tveit et al., 2015). The findings of this study underscore a significant novelty, suggesting that the previously observed efficacy of high-impact sports participation during adolescence in maintaining bone mineral density in later life may extend to a broader older population.

Interestingly, in the lumbar spine, no significant differences were observed in men. We hypothesize that the difference in lumbar spine aBMD transitions in men and women is a contributing factor. According to a previous study, lumbar spine aBMD in men plateaus after the age of 40 (Berger et al., 2008), whereas in women, it declines dramatically after menopause (Harris and Dawson-Hughes, 1992). Accelerated bone loss in women is a major difference from that in men. Therefore, we expect that women are more likely to differ from those with a high aBMD to those with a low aBMD because of the large changes associated with aging.

In this study, we focused our analysis on adolescence, adjusting for current exercise habits. However, we did not consider exercise habits spanning from adulthood to old age. It is plausible that individuals who engaged in high-impact sports like basketball or volleyball during adolescence might exhibit higher bone density in later years due to continued participation in similar activities. Indeed, several longitudinal studies have highlighted a correlation between adolescent physical activity and sustained activity levels into adulthood (Telama et al., 2005; Huotari et al., 2011). Notably, even in our preliminary analysis that accounted for limited data on exercise habits from the 20’s–50’s, the correlation between adolescent involvement in basketball or volleyball and bone density persisted. Consequently, to comprehensively understand these observations, a future long-term prospective cohort study tracking participants from adolescence to old age is warranted.

In this study, swimming was associated with lumbar aBMD in women. However, previous studies have reported that swimming without impact is not associated with improved aBMD (Tenforde and Fredericson, 2011; Gómez-Bruton et al., 2013). Thus, we reanalyzed our study excluding compression fractures. In accordance with International Society for Clinical Densitometry (ISCD) (Lewiecki et al., 2004), we excluded vertebral fractures or degenerative changes with a 1 standard deviation (SD) BMD from immediately adjacent vertebra (*n* = 331). Reanalysis results showed no significant difference in swimming (*β* = 0.043, *p* = 0.859), and only volleyball (*β* = 0.121, *p* < 0.01) was significantly associated with lumbar spine aBMD (Supplementary Table S7).

The current study had a few limitations. First, it is possible that there is a selection bias because this study included only participants living in urban wealthy areas of Japan. Second, we do not know the details of exercise intensity, amount of exercise, and duration. Also, there is no information on what sport he/she majored in, in which there are multiple sport types, such as skiing, skating, and track and field. Third, because we interviewed the participants about sports club activities from 50 to 60 years ago, a recall bias should be considered. However, in Japan, traditional sports club activities are performed in junior and high schools as part of education (Cave, 2004), and sports clubs are a major part of their school life; therefore, it is assumed that the recall bias is limited. Finally, since this study was cross-sectional, it was not possible to establish a causal relationship. Further prospective and intervention studies are needed in larger populations to clarify the causality between adolescent sport types and aBMD in older age.

In conclusion, both men and women who played adolescent basketball had higher femoral neck aBMD in old age. In addition, women (not men) who played adolescent volleyball had higher lumbar spine aBMD in old age. Therefore, playing relatively high-impact sports during adolescence may be beneficial for increasing aBMD later in life, even for non-elite athletes.

## Data Availability

Some or all datasets generated and/or analyzed during the current study are not publicly available; however, they can be obtained from the corresponding author upon reasonable request.

## Abbreviations

BM, bone mass; aBMD, areal bone mineral density; IPAQ, International Physical Activity Questionnaire; PBM, peak bone mass.
