# Corrigendum: Playing basketball and volleyball during adolescence is associated with higher bone mineral density in old age: the Bunkyo Health Study

**DOI:** 10.3389/fphys.2024.1385648

**Published:** 2024-03-27

**Authors:** Hikaru Otsuka, Hiroki Tabata, Huicong Shi, Mari Sugimoto, Hideyoshi Kaga, Yuki Someya, Hitoshi Naito, Naoaki Ito, Abulaiti Abudurezake, Futaba Umemura, Tsubasa Tajima, Saori Kakehi, Yasuyo Yoshizawa, Muneaki Ishijima, Ryuzo Kawamori, Hirotaka Watada, Yoshifumi Tamura

**Affiliations:** ^1^ Sportology Center, Graduate School of Medicine, Juntendo University, Bunkyo-ku, Tokyo, Japan; ^2^ Department of Sports Medicine and Sportology, Graduate School of Medicine, Juntendo University, Bunkyo-ku, Tokyo, Japan; ^3^ Department of Metabolism and Endocrinology, Graduate School of Medicine, Juntendo University, Bunkyo-ku, Tokyo, Japan; ^4^ Graduate School of Health and Sports Science, Juntendo University, Inzai-shi, Chiba, Japan; ^5^ Department of Healthy Life Expectancy, Graduate School of Medicine, Juntendo University, Bunkyo-ku, Tokyo, Japan; ^6^ Department of Medicine for Orthopaedics and Motor Organ, Graduate School of Medicine, Juntendo University, Bunkyo-ku, Tokyo, Japan; ^7^ Faculty of International Liberal Arts, Juntendo University, Bunkyo-ku, Tokyo, Japan

**Keywords:** bone mass, sports type, cross-sectional study, femoral neck, lumbar spine, exercise history

In the published article, the reference “Kannus, P., Haapasalo, H., Sankelo, M., Sievänen, H., Pasanen, M., Heinonen, A., et al. (1995). Effect of starting age of physical activity on bone mass in the dominant arm of tennis and squash players. *Ann. Intern Med.* 123 (1), 27–31. doi:10.7326/0003-4819-123-1-199507010-0003” was incorrectly included. It should have been “Hendrickx, G., Boudin, E., & Van Hul, W. (2015). A look behind the scenes: the risk and pathogenesis of primary osteoporosis. *Nat. Rev. Rheumatol.*, 11(8), 462–474. doi: 10.1038/nrrheum.2015.48.”

The authors apologize for this error and state that this does not change the scientific conclusions of the article in any way. The original article has been updated.

