# Supplementary material for: Playing basketball and volleyball during adolescence is associated with higher bone mineral density in old age: the Bunkyo Health Study
Source: Front Physiol. 2023 Oct 12;14:1227639. doi: 10.3389/fphys.2023.1227639 (PMC10602637; doi:10.3389/fphys.2023.1227639)
Supplement: Supplementary file 1 [file Table1.pdf]

Supplementary Table S1. Number of people engaged in each sport types during adolescence.

	Men (n=428)			Women(n=429)	
	n	(%)		n	(%)
Baseball and softball	99	23.1	Volleyball	148	34.5
Basketball	65	15.2	Tennis	87	20.3
Judo	49	11.4	Table tennis	72	16.8
Table tennis	46	10.7	Basketball	53	12.4
Tennis	45	10.5	Baseball and softball	38	8.9
Track & field	41	9.6	Gymnastics	32	7.5
Volleyball	37	8.6	Track & field	30	7
Swimming	29	6.8	Swimming	16	3.7
Soccer	23	5.4	Mountaineering	16	3.7
Mountaineering	20	4.7	Dance	13	3
Kendo	15	3.5	Ski and skating	6	1.4
Ragby	12	2.8	Badminton	4	0.9
Gymnastics	11	2.6	Kendo	3	0.7
Ski and skating	8	1.9	Kyudo	1	0.2
Boxing	6	1.4	Rowing	1	0.2
Kyudo	5	1.2	Golf	1	0.2
Karate	4	0.9			
Handball	4	0.9			
Wrestling	4	0.9			
ice hockey	4	0.9			
Badminton	3	0.7			
Rowing	3	0.7			
Golf	2	0.5			
Equestrian	2	0.5			
Aviation	1	0.2			
Weightlifting	1	0.2			
Sailing	1	0.2			
American football	1	0.2			

Supplementary Table S2. Comparison of aBMD across different levels of bone loading exercise groups considering exercise habits during adulthood.

Men	Non Sports	Repetitive non-impact	Repetitive low-impact	Odd-impact	High-impact	*P* value
	n=308	n=28	n=32	n=265	n=48	
Femoral neck aBMD	0.734 ± 0.006	0.723 ± 0.020	0.737 ± 0.018	0.732 ± 0.006	0.720 ± 0.015	*P=0.897*
Lumber spine aBMD	1.049 ± 0.011	1.100 ± 0.036	1.062 ± 0.034	1.088 ± 0.012	1.061 ± 0.027	*P=0.172*
Women	Non Sports	Repetitive non-impact	Repetitive low-impact	Odd-impact	High-impact	*P*-Value
	n=537	n=13	n=54	n=133	n=177	
Femoral neck aBMD	0.582 ± 0.004	0.580 ± 0.024	0.596 ± 0.012	0.587 ± 0.007	0.586 ± 0.006	*P=0.817*
Lumber spine aBMD	0.823 ± 0.006	0.830 ± 0.042	0.837 ± 0.020	0.822 ± 0.013	0. 851 ± 0.011	*P=0.286*

Supplementary Table S3. Associations between sports engaged in adolescence and femoral neck aBMD in older men considering exercise habits during adulthood.

Variables	Unadjusted β	Adjusted β	*P* value	Adjusted β 95%CI	
				Lower	Upper
Basketball	0.029	0.077	*0.037*	0.002	0.056
Rugby	0.051	0.060	*0.099*	-0.010	0.111
Tennis	0.025	0.055	*0.137*	-0.008	0.057
Soccer	0.003	0.005	*0.882*	-0.041	0.047
Table tennis	-4.20×10^{-4}	-0.001	*0.979*	-0.032	0.031
Baseball and softball	-0.001	-0.004	*0.916*	-0.024	0.022
Mountaineering	-0.004	-0.007	*0.855*	-0.051	0.043
Track & field	-0.004	-0.009	*0.813*	-0.037	0.029
Judo	-0.004	-0.010	*0.792*	-0.035	0.027
Swimming	-0.007	-0.013	*0.733*	-0.046	0.033
Volleyball	-0.007	-0.015	*0.677*	-0.042	0.027
Kendo	-0.026	-0.035	*0.341*	-0.080	0.028
Gymnastics	-0.031	-0.036	*0.329*	-0.094	0.032
Age (in years)	-0.001	-0.047	*0.224*	-0.003	0.001
Bodyweight (kg)	0.004	0.300	*<0.001*	0.003	0.005
Current smoking (n/%)	-0.016	-0.049	*0.194*	-0.040	0.008
Past Smoking (n/%)	0.003	0.011	*0.781*	-0.016	0.021
Years of education (n/%)	0.001	0.017	*0.649*	-0.003	0.004
Diabetes mellitus (n/%)	0.008	0.026	*0.474*	-0.013	0.028
Calcium intake (mg/day)	-6.87×10^{-6}	-0.018	*0.641*	-3.58×10^{-5}	2.21×10^{-5}
Alcohol intake (g/day)	4.47×10^{-4}	0.118	*0.002*	1.70×10^{-4}	0.001
25(OH)D (nmol/L)	0.002	0.085	*0.023*	2.58×10^{-4}	0.003
Taking osteoporosis medication (n/%)	-0.069	-0.053	*0.142*	-0.161	0.023
Current physical activity level (Mets·hour/week)	-2.75×10^{-5}	-0.014	*0.710*	-1.73×10^{-4}	1.18×10^{-4}
Exercise habits in adulthood	0.005	0.079	0.038	2.99×10^{-4}	0.010

Adjusted R^2 value 0.129

Supplementary Table S4. Associations between sports engaged in adolescence and femoral neck aBMD in older women considering exercise habits during adulthood.

Variables	Unadjusted β	Adjusted β	*P* value	Adjusted β 95%CI	
				Lower	Upper
Basketball	0.031	0.075	*0.012*	0.007	0.055
Baseball and softball	0.012	0.025	*0.408*	-0.016	0.040
Track & field	0.013	0.024	*0.416*	-0.018	0.044
Table tennis	0.008	0.023	*0.432*	-0.012	0.029
Volleyball	0.005	0.018	*0.544*	-0.011	0.020
Tennis	0.004	0.014	*0.651*	-0.015	0.024
Dance	0.005	0.006	*0.840*	-0.043	0.052
Mountaineering	-0.001	-0.002	*0.953*	-0.044	0.041
Swimming	-0.002	-0.002	*0.945*	-0.045	0.042
Gymnastics	-0.001	-0.003	*0.927*	-0.032	0.029
Age (in years)	-0.003	-0.195	*<0.001*	-0.005	-0.002
Bodyweight (kg)	0.005	0.380	*<0.001*	0.004	0.005
Current smoking (n/%)	0.012	0.023	*0.477*	-0.022	0.047
Past Smoking (n/%)	-0.013	-0.053	*0.114*	-0.030	0.003
Years of education (n/%)	1.40×10^{-4}	0.003	*0.919*	-0.003	0.003
Diabetes mellitus (n/%)	0.006	0.017	*0.582*	-0.014	0.025
Calcium intake (mg/day)	-4.07×10^{-7}	-0.001	*0.966*	-1.93×10^{-5}	1.85×10^{-5}
Alcohol intake (g/day)	1.00×10^{-4}	0.014	*0.652*	-3.35×10^{-4}	0.001
25(OH)D (nmol/L)	0.002	0.101	*<0.001*	0.001	0.003
Taking osteoporosis medication (n/%)	-0.001	-0.004	*0.904*	-0.017	0.015
Current physical activity level (Mets·hour/week)	-1.13×10^{-5}	-0.005	*0.870*	-1.46×10^{-4}	1.24×10^{-4}
Exercise habits in adulthood	0.005	0.067	*0.029*	4.97×10^{-4}	0.009

Adjusted R^2 value 0.224

Supplementary Table S5. Associations between sports engaged in adolescence and lumbar spine aBMD in older men considering exercise habits during adulthood.

Variables	Unadjusted β	Adjusted β	*P* value	Adjusted β 95%CI	
				Lower	Upper
Rugby	0.089	0.059	*0.112*	-0.021	0.199
Tennis	0.045	0.057	*0.134*	-0.014	0.105
Baseball and softball	0.025	0.044	*0.250*	-0.017	0.067
Judo	0.032	0.042	*0.263*	-0.024	0.088
Basketball	0.027	0.040	*0.289*	-0.023	0.077
Swimming	0.033	0.034	*0.364*	-0.039	0.105
Gymnastics	0.044	0.028	*0.454*	-0.071	0.158
Kendo	0.014	0.010	*0.784*	-0.085	0.112
Mountaineering	0.011	0.009	*0.801*	-0.075	0.097
Soccer	0.007	0.007	*0.861*	-0.073	0.088
Track & field	-0.003	-0.004	*0.915*	-0.064	0.057
Volleyball	-0.014	-0.016	*0.670*	-0.077	0.050
Table tennis	-0.017	-0.022	*0.559*	-0.075	0.041
Age (in years)	0.004	0.110	*0.005*	0.001	0.007
Bodyweight (kg)	0.007	0.290	*<0.001*	0.005	0.008
Current smoking (n/%)	-0.038	-0.065	*0.090*	-0.083	0.006
Past Smoking (n/%)	0.019	0.042	*0.274*	-0.015	0.053
Years of education (n/%)	-0.003	-0.036	*0.340*	-0.009	0.003
Diabetes mellitus (n/%)	0.032	0.061	*0.100*	-0.006	0.069
Calcium intake (mg/day)	-5.60×10^{-6}	-0.008	*0.835*	-5.85×10^{-5}	4.73×10^{-5}
Alcohol intake (g/day)	0.001	0.094	*0.013*	1.33×10^{-5}	0.001
25(OH)D (nmol/L)	0.001	0.018	*0.638*	-0.002	0.004
Taking osteoporosis medication (n/%)	-0.100	-0.043	*0.239*	-0.268	0.067
Current physical activity level (Mets·hour/week)	-7.17×10^{-5}	-0.020	*0.595*	-3.36×10^{-4}	1.93×10^{-4}
Exercise habits in adulthood	0.006	0.047	*0.223*	-0.003	0.015

Adjusted R^2 value
0.104

Supplementary Table S6. Associations between sports engaged in adolescence and lumbar spine aBMD in older women considering exercise habits during adulthood.

Variables	Unadjusted β	Adjusted β	*P* value	Adjusted β 95%CI	
				Lower	Upper
Volleyball	0.035	0.080	*0.010*	0.009	0.062
Swimming	0.070	0.057	*0.068*	-0.005	0.145
Basketball	0.024	0.034	*0.272*	-0.019	0.066
Table tennis	0.010	0.017	*0.570*	-0.026	0.047
Tennis	0.007	0.013	*0.675*	-0.026	0.040
Dance	0.016	0.012	*0.704*	-0.067	0.099
Baseball and softball	0.006	0.008	*0.799*	-0.042	0.055
Gymnastics	-0.003	-0.004	*0.901*	-0.056	0.050
Track & field	-0.016	-0.017	*0.578*	-0.070	0.039
Mountaineering	-0.047	-0.038	*0.216*	-0.121	0.027
Age (in years)	0.001	0.035	*0.289*	-0.001	0.003
Bodyweight (kg)	0.008	0.364	*<0.001*	0.006	0.009
Current smoking (n/%)	-0.029	-0.032	*0.346*	-0.089	0.031
Past Smoking (n/%)	-0.011	-0.026	*0.451*	-0.040	0.018
Years of education (n/%)	0.003	0.044	*0.173*	-0.001	0.008
Diabetes mellitus (n/%)	0.047	0.083	*0.007*	0.013	0.082
Calcium intake (mg/day)	-1.29×10^{-5}	-0.024	*0.444*	-4.59×10^{-4}	2.01×10^{-4}
Alcohol intake (g/day)	-1.63×10^{-5}	-0.001	*0.966*	-0.001	0.001
25(OH)D (nmol/L)	0.003	0.119	*<0.001*	0.002	0.005
Taking osteoporosis medication (n/%)	-0.012	-0.025	*0.420*	-0.040	0.017
Current physical activity level (Mets·hour/week)	-1.37×10^{-4}	-0.036	*0.253*	-3.72×10^{-4}	9.82×10^{-5}
Exercise habits in adulthood	0.002	0.017	*0.586*	-0.006	0.010

Adjusted R^2 value 0.182

Supplementary Table S7. Associations between sports engaged in adolescence and lumbar spine aBMD in older women in excluding compression fractures.

Variables	unadjusted β	Adjusted β	*P* value	Adjusted β 95%CI	
				Lower	Upper
Volleyball	0.050	0.121	*<0.001*	0.022	0.078
Basketball	0.040	0.064	*0.061*	-0.002	0.081
Table tennis	0.026	0.048	*0.156*	-0.010	0.063
Tennis	0.014	0.027	*0.429*	-0.020	0.047
Dance	0.024	0.020	*0.553*	-0.055	0.104
Gymnastics	0.011	0.014	*0.685*	-0.041	0.063
Baseball and softball	0.004	0.006	*0.863*	-0.045	0.054
Swimming	-0.008	-0.006	*0.859*	-0.092	0.076
Track & field	-0.019	-0.022	*0.509*	-0.076	0.038
Mountaineering	-0.070	-0.056	*0.100*	-0.153	0.013
Age (in years)	0.001	0.030	*0.427*	-0.001	0.003
Bodyweight (kg)	0.007	0.350	*<0.001*	0.005	0.008
Current smoking (n/%)	-0.018	-0.023	*0.546*	-0.076	0.040
Past Smoking (n/%)	-0.003	-0.008	*0.834*	-0.033	0.027
Years of education (n/%)	0.002	0.028	*0.434*	-0.003	0.007
Diabetes mellitus (n/%)	0.045	0.084	*0.015*	0.009	0.081
Calcium intake (mg/day)	-1.64×10^{-5}	-0.032	*0.354*	-5.12×10^{-5}	1.83×10^{-5}
Alcohol intake (g/day)	2.10×10^{-4}	0.019	*0.586*	-0.001	0.001
25(OH)D (nmol/L)	0.004	0.140	*<0.001*	0.002	0.006
Taking osteoporosis medication (n/%)	0.012	0.029	*0.403*	-0.016	0.039
Current physical activity level (Mets·hour/week)	-1.52×10^{-4}	-0.043	*0.207*	-3.90×10^{-4}	8.47×10^{-5}

Adjusted R^2 value 0.160
